# Editorial: Genetic Pleiotropy in Complex Traits and Diseases

**DOI:** 10.3389/fgene.2022.897383

**Published:** 2022-04-25

**Authors:** Qian Xu, Can Yang, Yu-Fang Pei

**Affiliations:** ^1^ Department of Epidemiology and Biostatistics, School of Public Health, Medical College of Soochow University, Suzhou, China; ^2^ Jiangsu Key Laboratory of Preventive and Translational Medicine for Geriatric Diseases, Medical College of Soochow University, Suzhou, China; ^3^ Department of Mathematics, The Hong Kong University of Science and Technology, Hong Kong, China

**Keywords:** genetic pleiotropy, multi-traits method, genome-wide association study, Mendelian randomization, complex disease

Genetic pleiotropy, the phenomenon of a single gene or a single genetic variant influencing multiple phenotypes, appears to be pervasive in human genome. Thousands of genome-wide association studies (GWASes) have identified hundreds of thousands of genetic variants associated with hundreds of complex traits or diseases. Watanabe et al. (2019) systematically analyzed more than 4,000 publicly available GWASes, indicating the presence of widespread pleiotropy at the levels of genes (63%) and SNPs (31%). Identification of pleiotropic effects may help to explain the observations for shared heritability and comorbidity among multiple complex traits and shed light on the underlying biological mechanisms for those traits. However, progress in the identification and understanding of genetic pleiotropic effects remains limited. Indeed, the associations of genetic loci with multiple traits observed in GWAS studies are cross-phenotype associations, which are distinguished from pleiotropic effects. Solovieff et al. (2013) described three broad categories of pleiotropy in the context of complex traits: biological pleiotropy, mediated pleiotropy and spurious pleiotropy.

In recent years, with the increasing publicly availability of GWAS summary statistics, a number of statistical methods have been developed to evaluate the genetic overlap between multiple traits and diseases. Existing multi-trait methods can be broadly divided into multivariate methods and univariate methods (Hackinger and Zeggini, 2017). Univariate methods combine single-trait GWAS summary statistics to detect pleiotropic genetic variants. On the contrary, multivariate methods analyze all of the interesting traits in a unified model, which often require that individual-level genotype and phenotype data for all traits analyzed (Hackinger and Zeggini, 2017). Although existing statistical methods are available for detecting pleiotropic effects, it is still very challenging for systematic characterization of pleiotropy. New methodologies are highly demanding for interpreting the results of cross-phenotypes associations.

In the research topic on “Genetic Pleiotropy in Complex Traits and Diseases,” eight original studies are collected. It mainly focuses on the field of novel methodology development, identification of novel pleiotropic loci and the pathogenic mechanism of gene polymorphism in complex diseases.

Specifically, three studies focus on the development of novel methodology for detecting multi-traits associations based on GWAS summary data. Liu et al. presented powerful procedures to jointly analyze multiple traits based on GWAS summary statistics. They first adopted the generalized Berk-Jones (GBJ) test and the generalized higher criticism (GHC) test to analyze multiple traits and then combined the *p*-values of GBJ, GHC and the existing MinP (the minimum of the *p*-values) test by the aggregated Cauchy association test, namely omnibus (OMNI) test. The proposed tests can well control the type I error rate and the OMNI test is robust to the signal directions, the correlation structures and the degrees of sparsity. Replication plays a key role in evaluating the credibility of pleiotropic effects. Ning et al. evaluated four methods for replicating multi-trait associations and introduced a four-level replication strategy. The described methods are implemented in the open-source R package MultiABEL. Fu at al. developed a hierarchical clustering approach (HCDC) to jointly analyze multiple phenotypes. Simulation and real data analyses demonstrated that existing methods embedding HCDC could improve power and uncover novel associated variants.

In the field of identifying pleiotropic loci for complex disease, three contributed papers identified several novel pleiotropic loci and revealed shared genetic components between complex traits. Lu et al. performed eQTL-weighted integrative analysis and produced a single *p*-value for the gene, and then utilized conjunction conditional FDR (ccFDR) approach to identify pleiotropic genes. They identified 14 genes commonly related to systemic lupus erythematosus (SLE) and rheumatoid arthritis (RA), four of which may be novel. Their study provided new insights into the shared genetic basis between RA and SLE. Similarly, using summary statistics from publicly available single-trait GWAS, R. Bellomo et al. conducted 15 separate multi-trait GWAS centered around coronary artery disease (CAD), peripheral artery disease (PAD) and seven atherosclerosis risk factors. They identified 25 novel pleiotropic loci which may elucidate novel genetic etiologies of atherosclerosis. In addition, Feng et al. showed that *ICAM-1*, a candidate gene associated with multiple cancers (e.g., breast cancer and gastric cancer), was associated with the risk of cervical cancer in the northern Chinese Han population.

The other two studies investigated common genetic risk mechanisms underlying multiple traits/diseases using Mendelian randomization (MR) analyses. Barowsky et al. performed a bi-directional MR analyses and a cross-disorder meta-analysis for osteoarthritis (OA) and major depression (MD), suggesting that OA and MD share common genetic risk mechanisms. Moreover, Yang et al. investigated the causal association of phospholipid fatty acids with breast cancer and prostate cancer using MR approach, but identified little evidence of causal association.

In conclusion, these articles included in this research topic comprehensively expounded the significance and challenges of genetic pleiotropy from the aspects of new methodology development, the identification of novel pleiotropic genetic loci and the molecular mechanisms underlying pleiotropic genes. We expect that improvements in statistical methods and biotechnology will promote more developments on this important topic.
